# Mini-Review: PDPK1 (3-phosphoinositide dependent protein kinase-1), An Emerging Cancer Stem Cell Target

**DOI:** 10.29245/2578-2967/2021/1.1194

**Published:** 2021-04-30

**Authors:** Bogdan Domrachev, Sitanshu Singh, Dandan Li, Udo Rudloff

**Affiliations:** 1 Rare Tumor Initiative, Pediatric Oncology Branch, Center for Cancer Research, National Cancer Institute, Bethesda, MD, USA; 2 Thoracic & GI Oncology Branch, Center for Cancer Research, National Cancer Institute, Bethesda, MD, USA

**Keywords:** Cancer Stem Cells, 3-Phosphoinositide Dependent Protein Kinase-1, Label Retaining Cancer Cells, Drug Resistance, Chemotherapy

## Abstract

Cancer stem cells (CSCs) are subpopulations of tumor cells that possess abilities for self-renewal, differentiation, and tumor initiation. These rare but therapy-recalcitrant cells are assumed to repopulate tumors following administration of systemic chemotherapy driving therapy failure, tumor recurrence, and disease progression. In early clinical trials, anti-CSC therapies have found limited success to-date possibly due to the inherent heterogeneity and plasticity of CSCs and the incomplete characterization of essential CSC targets. Here, we review the role of 3-phosphoinositide dependent protein kinase-1 (PDPK1) as an emerging CSC target. While most previous studies have relied on CSC models which are based on lineage and tissue-specific marker profiles to define the relationships between putative target and CSC traits, this review discusses PDPK1 and its role in CSC biology with an emphasis on CSC systems which are based on proposed function like label-retaining cancer cells (LRCCs).

## The cancer stem cell (CSC) hypothesis

The near universal development of resistance to systemic cytotoxic chemotherapy has been a stubbornly incalcitrant problem in oncology for decades. Commonly, after an initial period of tumor regression and disease control patients afflicted by advanced cancers experience disease progression, detoriation of their quality of life, and succumb to their illness^1^. Thus, the introduction of the CSC hypothesis, which provided a fundamentally novel explanation for the commonly observed treatment failures and raised prospects to overcome therapy resistance via novel therapy approaches, was greeted with great interest^2^.

**Figure 1.**
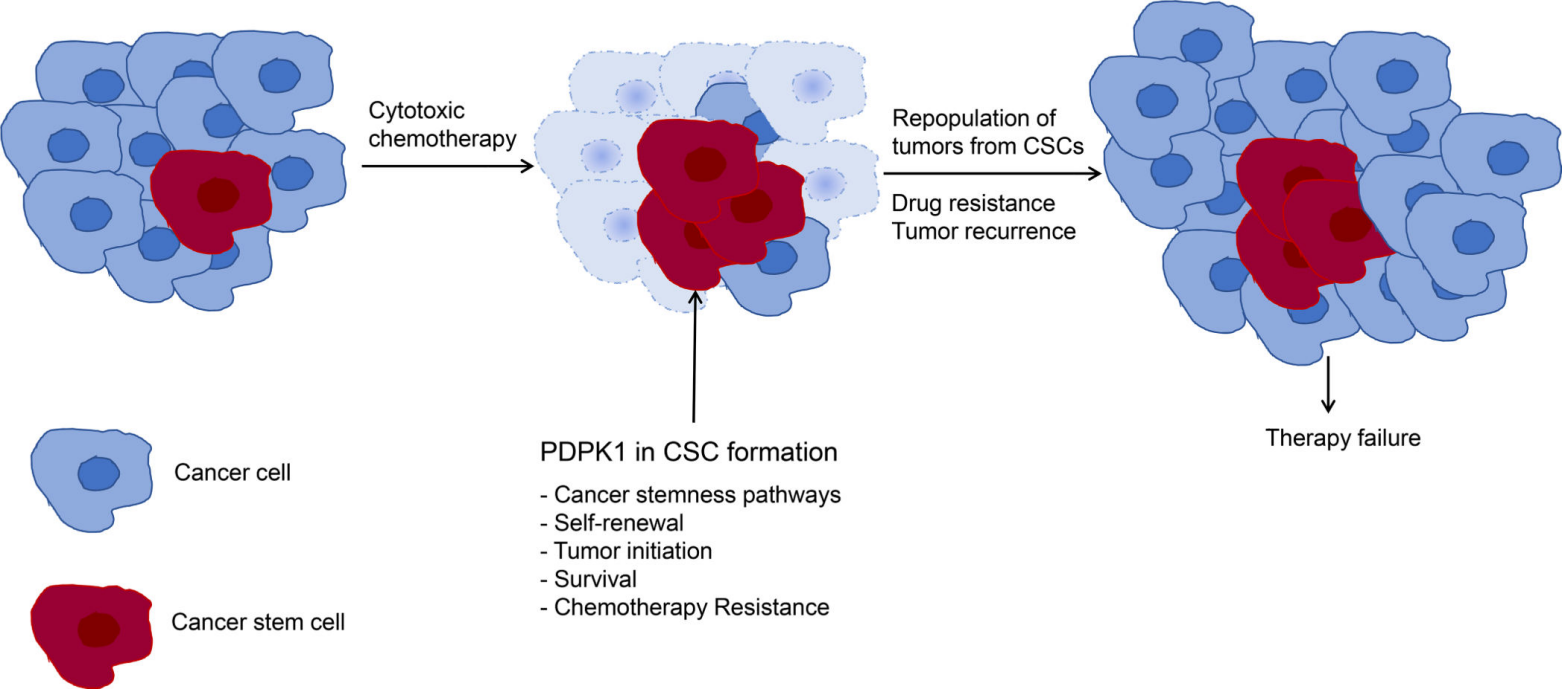

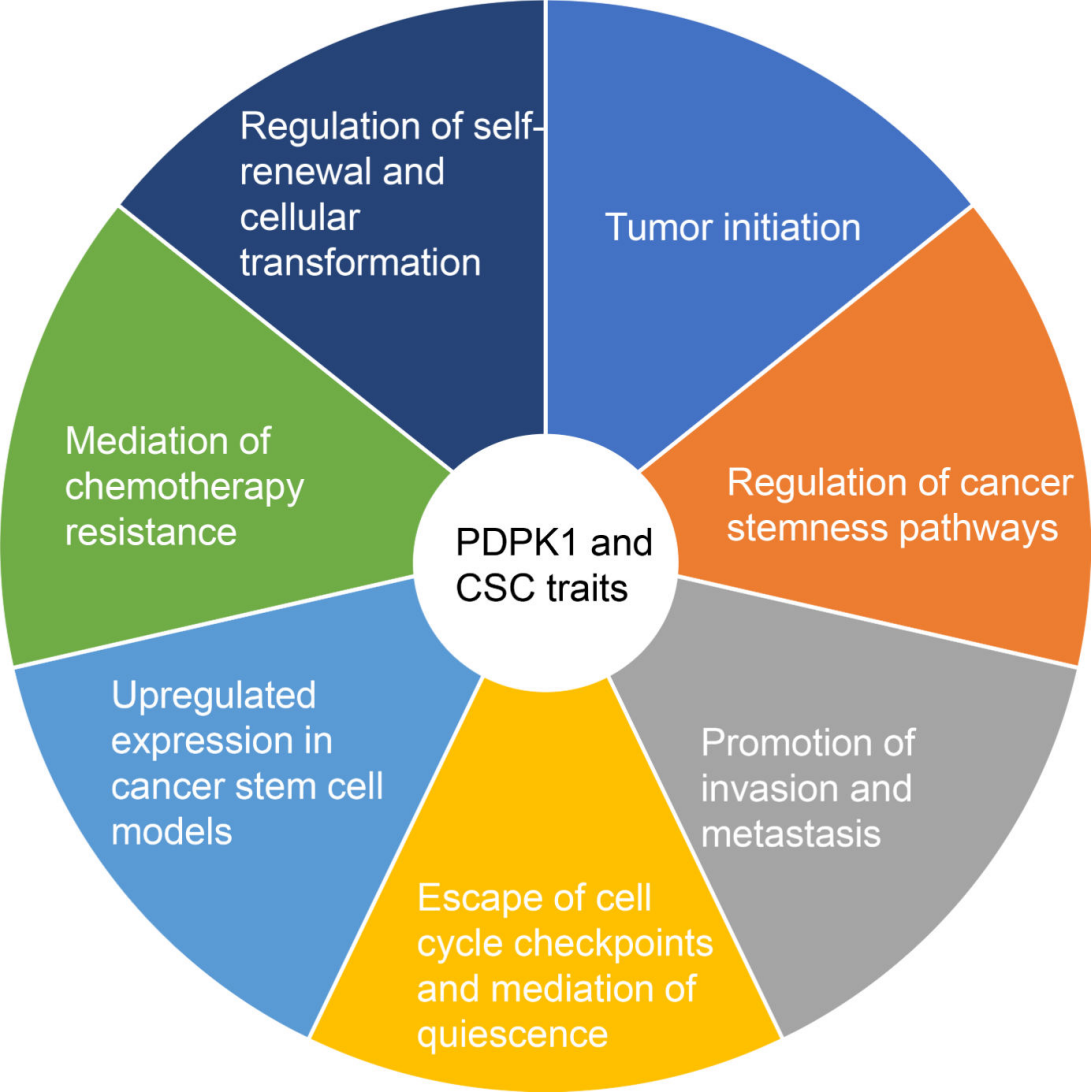
Role of CSCs in mediation of therapy resistance and tumor recurrence. **A**. Self-renewal of therapy-resistant CSCs repopulate tumors leading to therapy failure including cancer recurrence and disease progression. **B**. PDPK1 signaling involved in CSC function. CSC traits supported by PDPK1 are listed.

Three decades ago, the identification of tumor-initiating cells within hematological malignancies at the Princess Margaret Cancer Centre in Toronto, Canada^3,4^, and from breast cancer by Clark and colleagues thereafter^5^, suggested a hierarchical model of carcinogenesis^6^. Contrary to the clonal evolution model which suggests stochastic cancer cell divisions resulting in biologically equivalent cancer cells in each daughter cell generation, the CSC theory holds that (1) cancer arises from cells with dysregulated self-renewal mechanisms, and (2) cancer is comprised of a heterogeneous mass of cells which include a small fraction of stem-like progenitor cells that drive tumor progression, and that these cells are functionally distinct from the bulk, differentiated cancer cells^2,7,8^. While the exact origin of the CSC remains debated with experimental research supporting both CSCs arising via malignant transformation from normal stem cells as well as via de-differentiation of cancer cells^9,10^, the concept of CSCs does explain the inherent heterogeneity of tumors and the commonly observed resistance to chemoradiotherapy^2,11^ Failure to eradicate these self-renewing, resistant cells leads to repopulation of residual tumors postchemotherapy treatment with new cancer cells from this pool of cells and is cause for tumor recurrence (Fig. 1A)^12^. However, accepting an exclusively hierarchical model to capture cancer pathogenesis is at odds with several clinical findings: for most cancers, the concept of effective tumor burden reduction due to the assumed chemosensitivity of the bulk non-CSC tumor cell population is not observed in the clinic as tumor, in particular solid organ malignancies, not uncommonly do not regress at all upon treatment with systemic chemotherapy^1^. The concept of treatment-escaping CSCs, self-renewal and repopulating proliferating tumor cell progeny does not apply when tumor recurrence occurs very rapidly, or when cancer treatment accelerates tumor growth. The hierarchical model works best in clinical scenarios which initially see a reduction in tumor burden and then, after a latency period, tumors recur.

## Mechanisms of cancer stem cell therapy resistance – inherent versus acquired?

A plethora of in vitro, in vivo as well as findings in patients’ tumoral biopsies support that CSCs are inherently, *a priori*, resistant to chemoradiotherapy^8,14^. Inherent CSC characteristics mediating resistance to chemoradiotherapy include upregulation of DNA damage repair mechanism on multiple levels including upregulation of G_1_/M and G_2_/S checkpoints, efficient scavenging of reactive oxygen species (ROS), increased drug efflux via upregulation of ATP-binding cassettes and other drug transporters, or upregulation of pro-survival, anti-apoptosis regulators like Bcl-2 or Bcl-X_L_^8^. Conventional chemotherapies are most effective in rapidly proliferating cells going frequently through S phase. CSCs on the other hand are known to be quiescent cells which have left the cell cycle and thus escape the genotoxic effect of many currently employed chemotherapy agents^15,16^. Additionally, CSCs can effectively sense chemotherapy agents as xenobiotics and through activation of ALDH1A1 or ALDH3A1 enzymes which effectively can metabolize chemotherapy agents^17^. However, CSCs are also able to acquire under therapy pressure novel therapy resistance traits^18^. Following short courses of chemotherapy the fraction of CSCs to non-CSC tumoral bulk cells has been shown to change dramatically^14^. Comparing CSCs between chemotherapy-naïve versus chemotherapy-resistant conditions, CSCs acquired a more aggressive phenotype manifested by upregulation of the self-renewal factors NANOG, OCT4, and SOX2 as well as of ALDH3A1. Interestingly, effects on these stemness regulators differed between administered chemotherapeutics. The significant plasticity of CSCs adapting their phenotype under chemotherapy pressure is intriguingly also discussed in the origin and initial emerge of CSCs^20^. Recent findings show that therapeutic pressure can induce in non-CSCs a transient, stem-like state which is drug resistant due to the induction of EMT factors and other CSC traits. These therapy-induced CSC-like cells revert to their original phenotype upon withdrawal of chemotherapy underscoring the dynamic nature of their adaptive mechanisms in response to cancer therapy.

## Intratumoral CSC heterogeneity

CSC heterogeneity is, in large, also the result of the complex dynamic interplay and interdependence between CSCs and surrounding stromal milieu forming the CSC niche. One of the best investigated CSC niche–CSC interface are adaptations of CSCs residing in hypoxic areas^21^. Hypoxic, poorly perfused areas of solid organ cancers are most heavily populated with CSCs. Within these hypoxic niches CSCs create a unique environment by attracting M2-like tumor associated macrophages, suppressing dendritic and cytotoxic T cell function, upregulate inhibitory immune checkpoints or pro-angiogenic signals^21,22^. CSC residing in peri-vascular regions on the other hand find a nutrient-rich environment, have higher proliferative rates and shape vascularization and ECM formation^23^. CSCs within the invasive front of tumors have upregulated migratory and invasive capabilities, and the overlap with programs essential for metastasis like e.g. the CXCR4/ CXLX12 axis suggests these cells to be causally involved in the metastatic spread of cancers^21^. Overall, while the ‘niche concept’ is a simplification of the very complex and dynamic interplay of CSCs with nearly all stromal elements ECM components, it provides functional rationale for the phenotypic differences and heterogeneity observed among CSCs. As CSCs need to find their niches, vice versa, they become major educators of their microenvironment. Reciprocally, these educated stromal cells signal back and impact CSC traits like CSC quiescence, self-renewal, or therapy resistance^21^.

It is also likely that cancer stem cells are coming in and out of the CSC pools increasing heterogeneity and pleiotropism of CSC traits within tumors further^9^. In this regard, targeting rare, infrequent CSC populations defined by CSC surface markers and an overreliance on such markers to capture CSC populations mediating chemotherapy resistance might have led to the largely disappointing early clinical results of anti-CSC therapy so far^7^. Thus, there has been a renewed refocus on preclinical CSC models with improved recapitulation of tumoral stem cell biology, like LRCCs, which are less biased by culture conditions, cell lineage markers, or results derived from immune compromised mouse models^9^.

Here we discuss the role of PDPK1 as an emerging regulator of cancer stemness by reviewing recent findings in functional CSC models like label-retaining cells (LRCCs), studies in autochthonous animal models of cancer, as well as CSC traits supported by PDPK1 (Fig. 1B).

## Regulation of cancer stemness pathways by PDPK1

PDPK1 is a phylogenetically conserved member and master regulator of the large AGC kinase family^24^. PDPK1 possesses a N-terminal kinase and a C-terminal pleckstrin homology (PH) domain which senses PI3K-generated phospho-inositide metabolites, in particular phosphatidylinositol (3,4,5)-trisphosphate (PtdIns(3,4,5) P3); PIP3), at the inner plasma membrane. The N-terminal kinase domain consists of the activation loop and the PDPK1-interacting fragment (PIF)-pocket which binds to a hydrophobic motif on PDPK1 substrates^25^. PDPK1 autophosphorylation of the activation loop (at serine 241) activates PDKP1^26^. Signaling output of constitutively activated PDPK1 is primarily determined by the pre-activation state and post-translational modifications of its substates as well as interactions with other protein modulators like the inhibitory 14-3-3 or the tumor suppressor candidate 4 (TUSC4) proteins^24,27,28^. Whereas AKT activation by PDPK1 is regulated via simultaneous PIP3 binding enhancing proximity and phosphorylation at threonine 308 of the activation loop of AKT, other AGC kinase substrates of PDPK1 like serum and glucocorticoid-regulated kinase 1 (SGK1), p70 ribosomal S6 kinase (p70S6K), protein kinase C (PKC), or p90RSK interact with PDPK1 through their PIF-binding motifs. Post-translational modifications of the PIF domains of these substrate kinases very heavily impact the affinity of PDPK1-kinase interactions and therewith PDPK1 signaling output. Thus the varying pre-activation states of PDPK1 substrate kinases explain the heterogenous and pleiotropic PDPK1 output across different cell lineages and the not uncommonly observed lack of correlation of PDPK1 activity and AKT activation^29,30^.

**Figure 2.**
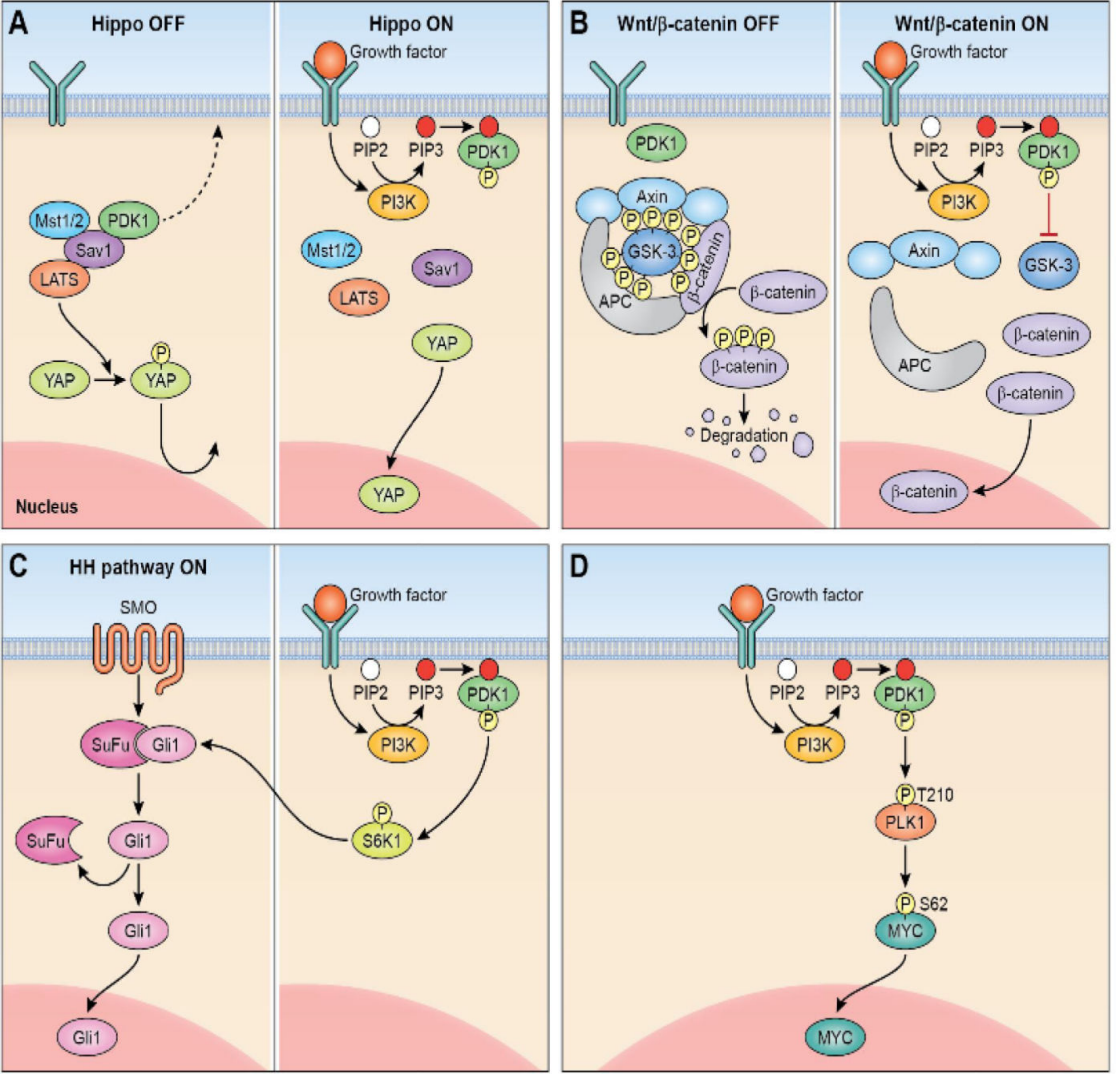
Regulation of cancer stemness pathways by PDPK1. **A**. Membranous recruitment of PDPK1 (right) activates Hippo pathway. **B**. Activation of WNT/β-catenin signaling. **C**. PDPK1 activation of S6 kinase activates hedgehog pathway (HH, hedgehog; SMO, smoothened). **D**. PDPK1-PLK1-MYC signaling axis in cancer.

Considering the pleiotropism of PDPK1 output, it is not surprising that PDPK1 was found to be an essential regulator of cancer stem cell signal transduction. Best examined is the Hippo signaling pathway^31^; in its non-activated form, PDPK1 forms in the cytoplasm a complex with Sav1 which allows the Hippo pathway components MST1/2 and LATS to phosphorylate YAP retaining this master regulator in the cytoplasm^32,33^. Following phosphorylation and membranous recruitment of PDPK1, this complex dissolves, YAP is allowed nuclear entry and activation of the YAP/TAZ stemness program occurs. Constitutively activated PDPK1 has been shown to stimulate β-catenin/ Wnt signaling in medulloblastoma where PDPK1 small molecule inhibition increased survival and enhanced the cytotoxic effects of chemotherapeutic drugs^34^. PDPK1-mediated activation of the AGC kinase S6K1 connects PDPK1 signaling output with the hedgehog pathway in cancer cells^35^. Recently, PDPK1 has also been shown to function as an upstream regulator of the cancer stemness master regulator Myc^36^. A summary of PDPK1-mediated regulation of cancer stemness signaling pathways is shown in Fig. 2.

## Cancer stem cell self-renewal and tumor initiation

PDPK1 has recently been identified as an upstream regulator of the c-Myc oncogene. PDPK1 can phosphorylate PLK1 on threonine 210 which, once phosphorylated, stabilizes Myc from proteasomal degradation via serine 62 phosphorylation^36,37^. Increased cellular levels of c-Myc promote cancer cell self-renewal, survival, and increased CSC fractions in tumors as loss-of-function experiments resulted in loss of CSCs and CSC functions upon silencing of PDPK1^36^. Of note, PDPK1-governed increased stability of c-Myc was associated with resistance to targeted therapy^36,38^. That PDPK1-governed CSC function can translate into enhanced tumor initiation *in vivo* has recently been elegantly shown in autochthonous pancreas cancer models. Eser and colleagues showed that hat PDPK1 is an essential effector of Kras, and that an intact PDPK1/PI3K axis is an essential tumor initiating event in cooperation with KRAS for increased cell plasticity, acinar-to-ductal metaplasia (ADM), and pancreatic ductal adenocarcinoma (PDAC) formation^39^. Similar findings of PDPK1 promoting tumor initiation in cooperation with Erbb2 or Ras activation have been made in breast cancer cells^40,41^, or in BRAF V600 mutant melanoma where loss of PDPK1 reduced tumor formation^42^.

## Mediation of resistance to chemotherapy

While there are many studies linking PDPK1 expression level and PDPK1 activation to chemoresistance^43^, it is not always clear that increased chemoresistance is due to PDPK1-promoted cancer stemness. AKT signaling with its pro-survival, anti-apoptosis functions is, for example, a well-known mediator of chemoresistance in cancer cells independent of tumor cell differentiation state and CSC function. Thus, one of the more compelling examinations of PDPK1-governed cancer stemness mediating resistance to chemotherapy emanates from studies in label-retaining cancer cells (LRCCs). LRCCs are slowly cycling, quiescent cells holding onto intracellular dyes^16^. Slow cycling LRCCs exhibit cancer stem cell and pluripotency traits and represent a distinct subpopulation of the heterogeneous CSC pool^44,45^. LRCCs have been shown to be more tumorigenic, mediate therapy resistance, and promote tumor recurrence^15,46,47^. Treatment with chemotherapy dramatically can increase the fraction of LRCCs^48^. It is believed that LRCCs undergo asymmetric cell divisions with non-random chromosomal co-segregation^45^. LRCCs provide a unique opportunity to study cancer stemness within a live, cell-based system that relies on stemness function rather than on marker profiles. Studying label-retaining pancreas cancer cells, Li and coworkers showed that PDPK1 is significantly upregulated in LRCCs compared to the non-LRCC fraction and that PDPK1 regulates survival and response to chemotherapy treatment in LRCCs, a finding also made by another group^47,49^. Findings that the stemness cell population survives chemotherapy and is the nidus for re-growth and disease relapse have also been made in acute myeloid leukemia (AML) where PDPK1 was found to be a major regulator of leukemia stem cell survival^50^.

## Conclusions and Future Perspectives

PDPK1 is an emerging CSC target in at least some cancer histologies. Therapies aimed at the eradication of CSCs involved in chemoresistance, repopulation of tumors, and cancer recurrence appear to be a logical translation of the cancer stem cell hypothesis into the clinic. While PDPK1 small molecule inhibitors have entered clinical testing it remains to be seen whether the therapeutic window targeting PDPK1 in CSCs versus unwarranted off-target effects on PDPK1 signaling essential in many physiologic processes is large enough for anti-PDPK1 targeted therapy to be safe in patients afflicted by cancer and become clinically feasible.
